# Perceptions about medical male circumcision and sexual behaviours of adults in rural Uganda: a cross sectional study

**DOI:** 10.11604/pamj.2015.22.354.7125

**Published:** 2015-12-11

**Authors:** Trasias Mukama, Rawlance Ndejjo, Geofrey Musinguzi, David Musoke

**Affiliations:** 1 Department of Disease Control and Environmental Health, School of Public Health, Makerere University College of Health Sciences,Kampala, Uganda

**Keywords:** Perceptions, circumcision, sexual behaviours, HIV/AIDS, prevention

## Abstract

**Introduction:**

Medical male circumcision is currently recognized as an additional important HIV preventive intervention to reduce the risk of heterosexually acquired HIV infection in men. However, sexual behaviours after medical circumcision can potentially reduce the expected benefits of the practice. This study explored the perceptions about medical male circumcision and sexual behaviours of adults in Kayunga district, Uganda.

**Methods:**

A cross-sectional study was carried out among 393 respondents using a semi structured questionnaire. In addition, four focus group discussions were conducted. Quantitative data was analysed using STATA 12. Univariate, bivariate and multivariate analyses were carried out. Qualitative data was analysed thematically.

**Results:**

The study established various perceptions about medical male circumcision and sexual behaviours. Majority 247 (64.5%) did not perceive circumcision as a practice that can lead men to have multiple sexual partners. Males were 3 times more likely to think that circumcision would lead to having multiple sexual partners than females (AOR=2.99, CI: 1.93-4.61). Only 89 (23.2%) believed that circumcision would lead to complacency and compromise the use of condoms to prevent against infection with HIV. Respondents who had education above primary were less likely to think that circumcision would compromise the use of condoms (AOR=0.49, CI: 0.31- 0.79). The perception that circumcised youths were less likely to abstain from sexual intercourse was less held among those with education above primary (AOR=0.58, CI: 0.37-0.91) and those older than 30 years (AOR=0.59, CI: 0.38-0.92).

**Conclusion:**

There were gaps in knowledge and negative perceptions about MMC in the study community. Measures are needed to avert the negative perceptions by equipping communities with sufficient, accurate and consistent information about medical male circumcision and sexual behaviour.

## Introduction

Despite growing efforts in biomedical research towards HIV/AIDS prevention and control, there is slow progress towards reducing HIV infection rates [1]. One key intervention in the fight has been the addition of medical male circumcision (MMC) as part of the comprehensive HIV prevention package [2] following evidence that it is efficacious in reducing sexual transmission of HIV from women to men [3–5]. In Eastern and Southern Africa where HIV prevalence is high, mathematical modeling [6] shows that circumcising 80% of males aged 15-49 in 5 years can avert 3.4 million infections [7]. Post MMC sexual behaviours of both males and females can potentially reduce the expected benefits of the practice. The perceptions that communities hold about MMC are likely to affect their sexual behaviours after undergoing the procedure. Men might feel less inhibited about engaging in risky sexual behaviours [8]. Likewise, women might be complacent about using other prevention methods like condoms [8]. Several studies have been conducted to relate male circumcision and its influences on sexual behaviours. The three randomized controlled trials that investigated the efficacy of male circumcision for HIV prevention also investigated their sexual behaviours after circumcision. In the trial conducted in Uganda, there was no evidence of increased risky sexual behaviours among circumcised men [5]. In the South Africa trial, circumcised men reported having more sex compared to the uncircumcised men [4] while in the Kenya trial, circumcised participants reported more unprotected sex acts than their uncircumcised counterparts [3]. In another South African qualitative study amongst women, most thought that MMC would increase females’ risk of contracting HIV as circumcised men may engage in risky behaviours like having more sexual partners and not using condoms [8]. Another study that investigated MMC and how it would affect sexuality and sexual satisfaction established that the perceptions of communities and individuals about MMC affect the circumcision uptake rates and the post-circumcision sexual behaviours [9]. The success of MMC interventions is thus dependent upon post circumcision sexual behaviours which are influenced by perceptions. This study explored the perceptions about medical male circumcision and sexual behaviours of adults in Kayunga district, Uganda.

## Methods

**Study design:** a cross-sectional study that involved the use of quantitative and qualitative data collection tools was conducted. The study population comprised of females and both circumcised and un-circumcised males. A pretested questionnaire was used to collect quantitative data from 393 respondents aged between 18 and 60 years. The sample size was obtained using the formula by Kish Leslie [10] where p=0.5, z=1.96 and e=0.05. In addition, four focus group discussions were conducted to generate qualitative data. The focus group discussions were carried out separately for married men, married women, single men and single women.

**Study area:** the study was conducted in Kayunga district located in the central region of Uganda. It was the first district to launch mass male circumcision aimed at reducing the spread of HIV. Kayunga is approximately 74 kilometers northeast of Kampala, the country's capital city. The district is predominantly rural and subsistence agriculture is the main economic activity with emphasis on food crops. According to the 2014 national population census provisional results, Kayunga district had a total population of 370,210 people, of whom 180,541 were males and 189,669 were females. The population growth rate in the district is 1.9% [11].

**Sampling:** simple random sampling was used to select 4 sub-counties out of the 9 in the district. From each sub county, 2 parishes were selected from which 2 villages were then obtained. Systematic random sampling was used to select the households that participated in the study. The sampling interval, which ranged from 3 to 5, was determined by the approximate number of households in each village. From each household, one participant (either the household head or their spouse) responded to the questionnaire.

**Data collection:** the study questionnaire and focus group discussion guides were developed in English and translated to Luganda, the main local language in Central Uganda. Research assistants were trained on questionnaire administration and other pertinent research procedures. Focus group discussions were recorded using a tape recorder.

**Data entry and analysis:** quantitative data was entered and cleaned in Epi Info 7.0 (Epi Info™ 7, CDC, Atlanta, USA) and analysed in STATA 12 (Stata Corp, Texas, USA) statistical software. Univariate analyses including frequency distributions were run to characterize the sample. Bivariate analysis was done to obtain significant associations between socio-demographics and various perceptions on sexual behaviours. To obtain a binary outcome variable for use in logistic regression, “ No” and “Not certain” were merged. A multivariable binary logistic regression model was used to identify the independent predictors of the various perceptions about post circumcision sexual behaviours. The perceptions on having multiple sexual partners, compromise of condom use, increase in sexual drive and decreased abstinence from sex among youths formed the outcome variables. These were run against the independent variables and associations found with a p-value of less than 0.05 were considered statistically significant. To get the adjusted odds ratios, potential confounders of age, sex, education levels and religion were adjusted for in the model. All focus group discussions were recorded and then transcribed verbatim in Luganda. Transcribed data was then translated into English. The transcripts were reviewed several times and by using a qualitative thematic analysis, data were coded into initially predetermined themes and other emerging themes. A second researcher reread the data to ensure coding had been done adequately as well as correctly assigning coded data to appropriate themes. The two researchers then harmonized the identified discrepancies to ensure the qualitative analysis was adequate. Some of the findings from the FGDs are presented verbatim in quotations.

**Ethical considerations:** ethical approval to carry out the study was obtained from the Makerere University School of Public Health, Higher Degrees, Research and Ethics committee. Permission to conduct the study was obtained from the District Health Office and the local/administrative leaders of Kayunga before commencement of the study. The study aims, benefits and potential risks were duly explained to all respondents before they provided written informed consent to participate in the study.

## Results

Socio**Socio-demographic characteristics of respondents:** the respondents comprised of 158 (40.2%) females and 235 (59.8%) males. The average age of respondents was 31.2 years (range 18-60) and more than half 217 (55.2%) were aged ≤30 years. Among the males, 150 (63.8%) were circumcised, 76 (50.7%) having undergone the procedure from health facilities. Most respondents were Christians 308 (78.4%), married 275 (70.0%) and had attained primary education or less 247 (62.8%). There was a significant difference in occupation among males and females (p=0.024) (Table 1).

**Table 1:** Socio-demographic characteristics of respondents by sex

Variable	Total	Male	Female	p-value
	Frequency (%)	Frequency (%)	Frequency (%)	
Total	393 (100.0)	235 (100.0)	158 (100.0)	
**Age**				
Below 30 years	217 (55.2)	129 (54.9)	88 (55.7)	
Above 30 years	176 (44.8)	106 (45.1)	70 (44.3)	0.875
**Educational level**				
Primary and below	247 (62.8)	140 (59.6)	107 (67.7)	
Above primary	146 (37.2)	95 (40.4)	51 (32.3)	0.101
**Religion**				
Christians	308 (78.4)	182 (77.4)	126 (79.8)	
Muslims	85 (21.6)	53 (22.6)	32 (20.2)	0.587
**Marital status**				
Married	275 (70.0)	157 (66.8)	118 (74.7)	
Others	118 (30.0)	78 (33.2)	40 (25.3)	0.095
**Occupation**				
Farming	179 (45.6)	118 (50.2)	61 (38.6)	
Others	214 (54.4)	117 (49.8)	97 (61.4)	0.024+
**Circumcision status**				
Not circumcised	150 (63.8)	150 (63.8)	-	
Circumcised	85 (36.2)	85 (36.2)	-	

+ Statistically significant at p < 0.05

**Knowledge and perceptions on medical male circumcision:** the majority 383 (97.5%) of respondents had heard about MMC. The main sources of information about MMC were radio 196 (49.9%) and health centres 141 (35.9%). Other sources of information included family and friends 99 (25.2%), posters 73 (18.6%), television 25 (6.4%) and newspapers 13 (3.3%). The main reasons for circumcision were HIV prevention 336 (85.5%), prevention of other STIs 299 (76.1%), improved penile hygiene 245 (62.3%), reduced cancer risks 15 (3.8%), treatment of certain health conditions 25 (6.4%) and sexual appeal 29 (7.4%). In addition, the FGDs revealed that incentives and economic conditions also influenced the decision of males to undergo the procedure. Some of the motivations to become circumcised were respect from peers, material benefits, and sexual appeal as supported by quotes from FGDs. *“I used to live among a community in Eastern Uganda where they could not give you any respect if you were not circumcised therefore one would end up getting circumcised just to fit in the society. ”* (Male participant) *“In some places where incentives like money, t-shirts and edibles are given, many youth go for circumcision not because they want but because of what is offered and when you look at the economic situation in our area, people are poor and can easily be lured into circumcision by simple incentives. ”* (Male participant) More than half 206 (53.8%) of the respondents believed that MMC could prevent a man from acquiring HIV. A total of 39 (10.2%) thought that MMC provided 100% protection against HIV acquisition, 291 (76.0%) thought it did not, while 53 (13.8%) did not know. Among those who said MMC offered partial protection, 145 (65.9%) reported protection of 50-70%, 55 (25.0%) mentioned between 5-45%, 20 (9.1%) said it offered 71-90% protection while 70 (24.1%) could not estimate. Regarding the recommended time between circumcision and resumption of sexual intercourse, the majority 226 (59.0%) of respondents mentioned between 6-12 weeks, whereas 127 (33.2%) said between 1-5 weeks. The rest 30 (7.8%) mentioned more than 13 weeks. The mean time between circumcision and resumption of sexual intercourse was 8 weeks (range 1-52 weeks). The majority 371 (97.1%) of respondents said they would circumcise their male children. Among the uncircumcised males, 68 (80%) would undergo circumcision if they had a chance. These gave STI/HIV prevention 36 (58.1%), hygiene 20 (32.3%), culture 4 (4.8%) and sexual appeal 2 (3.2%) as their reasons for willingness to be circumcised. The reasons why uncircumcised males would not want to be circumcised were: fear of pain (46.1%), age (15.4%), taking a long time to heal (9.0%), religious reasons (7.7%), unwilling family members (7.7%), did not understand circumcision (7.7%), or did not believe in the intervention (6.4%). The majority 367 (96.1%) of respondents would recommend their friends for circumcision. Most of the female respondents 145 (95.4%) would prefer their partner circumcised. Females who preferred circumcised partners cited HIV/STI prevention (66.1%), penile hygiene (29.2%), and religion (2.3%) as the main reasons. Females who did not want their partners circumcised cited the following reasons: old age, fear of their partners becoming polygamous, the time between circumcision and resumption of sexual intercourse being too long which could result in women diserting their husbands, and fear of infections that may be acquired in the process as also noted in the FGDs. “I do not trust the equipment used for circumcision. Sometimes people circumcised at the health facility are so many and you really can't be certain whether some equipment is not reused. As you know, there is a lot of corruption in our country and the officials might divert funds for buying equipment to personal use and they end up buying only a few and reusing them. ” (Male participant) “There is a possibility of a man acquiring infections during circumcision. In addition, the healing process takes a long time and the stitches keep breaking which all worries me. I would therefore not recommend my husband to be circumcised.” (Female participant)

**Perceptions about medical male circumcision and sexual behaviours:** the study revealed various perceptions about MMC and sexual behaviours. Some respondents perceived that MMC increases multiple sexual partnerships 136 (35.1%) and sexual drive 87 (22.7%). Almost a quarter of respondents believed that MMC would compromise condom use 89 (23.2%) and reduce likelihood of abstinence among youths 96 (25.1%). Only 41 (26.8%) of the females believed they would be less likely to demand for use of condoms with a circumcised man compared to when he was uncircumcised. From the FGDs, there were concerns about promoting MMC as a measure to reduce HIV infection and the sense of protection provided by the procedure. It was generally thought that the sense of protection offered by circumcision could lead men and women to exhibit less restraint sexual behavior as supported by the quotes below. *“Promoting circumcision for reducing the risk of HIV acquisition is a problem. If I have been using condoms and you tell me that my risk of acquiring HIV is reduced because of being circumcised, I don't think I would use a condom any more. Imagine we were playing football and i am wearing boots while you are barefooted. Do you really think we would attack an opponent in the same way? It definitely can't happen because you would be aware of your vulnerability to injury unlike me who would afford to take more risks.”* (Male participant) *“I would not want to have my man circumcised because circumcision would increase his sexual drive and he would marry more women. Equally worrying is the time between circumcision and resumption of sexual intercourse which is very long and I doubt whether he can abstain for such a time.”*(Female participant) *“Circumcised men may stop using condoms thinking that they are protected. They also have the desire to show their partners that they are circumcised and are thus less willing to use condoms.”* (Female participant) *“I have seen many men who get circumcised. Even those who were initially not sexually active tend to change after being circumcised which indicates that circumcision increases their sexual drive.”* (Male participant) Males were almost three times more likely to perceive that men would have multiple sexual partners following circumcision (AOR=2.99, CI: 1.93- 4.61). Respondents with education above primary were less likely to think that circumcision would compromise the use of condoms (AOR= 0.49, CI: 0.31- 0.79) and lead to youths not abstaining from sex (AOR= 0.58, CI: 0.37- 0.91). Muslims were 1.73 times more likely to think that circumcision would increase the sexual drive of men (AOR=1.73, CI: 1.07- 2.84) (Table 2).

**Table 2:** Adjusted odds ratios for perceptions on post circumcision sexual behaviours

Variable	Having multiple sexual partners		Compromising condom use		Increase in sexual drive		Circumcised youth less likely to abstain	
	AOR (95% CI)	p-value	AOR (95% CI)	p-value	AOR (95% CI)	p-value	AOR (95% CI)	p-value
**Sex**								
Female	1		1		1		1	
Male	2.99[1.93-4.61]	**<**0.001+	1.04[0.65-1.66]	0.88	0.98[0.64-1.50]	0.926	0.89[0.57-1.39]	0.606
**Age**								
Below 30 years	1		1		1		1	
Above 30 years	0.82[0.53-1.25]	0.355	0.66[0.41-1.05]	0.077	1.21[0.80-1.85]	0.365	0.59[0.38-0.92]	0.019+
**Educational level**								
Primary and below	1		1		1		1	
Above Primary	0.58[0.37-0.91]	0.017+	0.49[0.31-0.79]	0.003+	0.79[0.51-1.23]	0.296	0.58[0.37-0.91]	0.017+
**Religion**								
Christians	1		1		1		1	
Muslims	1.43[0.84-2.40]	0.185	1.57[0.87-2.84]	0.137	1.73[1.07-2.84]	0.027+	1.23[0.73-2.17]	0.403
**Marital status**								
Married	1		1		1		1	
Others	1.01[0.62-1.63]	0.978	0.71[0.42-1.18]	0.188	1.27[0.79-2.04]	0.322	0.90[0.54-1.48]	0.677
**Occupation**								
Farming	1		1		1		1	
Others	0.98[0.61-1.56]	0.924	1.33[0.80-2.22]	0.27	0.80[0.51-1.26]	0.339	1.06[0.65-1.71]	0.816
**Circumcision status**								
Not circumcised	1		1		1		1	
Circumcised	1.14[0.61-2.13]	0.682	1.12[0.59-2.16]	0.723	1.28[0.69-2.40]	0.435	1.10[0.59-2.06]	0.767

+ Statistically significant at p < 0.05

## Discussion

The level of knowledge of respondents on the benefits of MMC was high. This could be attributed to the various MMC promotion campaigns ongoing in the entire country. Listening to radio and visiting health centres were the main sources of information about MMC. Indeed, mass media is a key source of health information for influencing behaviour for most people living in Africa [12, 13]. This is possibly due to their high accessibility and affordability. However, there were knowledge gaps about certain aspects of MMC in this study. These included the level of protection against HIV acquisition, and time lag between circumcision and resumption of sexual intercourse. This portrays the existence of inconsistent and inaccurate information concerning MMC which could have negative effects on the community. Similar to a study conducted in Kenya, few participants were able to accurately state the percentage reduction in HIV risk [14]. Since the major source of information on MMC in this study was radio, the knowledge gap could be attributed to media framing as this has been found to shape people's knowledge, attitudes and beliefs about a particular topic [15]. There is therefore need to ensure that MMC clients and their partners receive accurate and consistent information about MMC. This should emphasize; the fact that MMC only offers partial protection against HIV acquisition, the recommended period of abstinence following circumcision and the need to use other HIV prevention methods.

The acceptability of MMC was generally high in this study. It was higher for females (95.4%) compared to males (80%). Other studies have also indicated that acceptability for MMC is high amongst sub-Saharan African men and women and is also acceptable for children [16–20]. Prevention of STIs and improved penile hygiene were the major reasons for MMC acceptability. This is similar to the findings from other studies [16, 19, 21, 22]. Many people thus believed in MMC and its benefits and would undergo the procedure. The main barriers to circumcision included pain, old age, and uncertainty of safety of instruments. These are similar to the ones reported by previous acceptability studies [16, 23–26]. The circumcision prevalence rate in Uganda is 26% [27] while MMC acceptability rate of uncircumcised men for their male children is 69-86% in central Uganda [19]. The existence of barriers identified in this study could be responsible for the difference that exists between acceptability levels and circumcision prevalence rates [16]. There is therefore need to reduce the barriers affecting uptake of MMC if the practice rates are to be reflective of the acceptability rates. This study found that respondents with post primary education were 2 times less likely to hold the perception that circumcision compromises the use of condoms. The perception could be due to differences in access and awareness of other HIV prevention methods. For instance, the 2011 Uganda AIDS Indicator survey found that youth with higher educational attainment were more likely to have used a condom at first intercourse compared to their less educated peers [27]. Therefore, access to other HIV prevention methods might lead more educated community members to perceive MMC as unnecessary and would affect their existing prevention behaviours. People with lower education might view MMC as a cheaper method of HIV prevention due to lack of access to alternative HIV prevention methods. This could also be due to the existence of inaccurate information about MMC benefits in communities most especially regarding the level of protection it offers. The views about the protective role of MMC could have influenced the perception that it would affect use of condoms. This perception may not lead to negative post-circumcision practices. Indeed there was no statistically significant difference between circumcised and uncircumcised respondents as regards the practice.

Males were more likely to hold the perception that MMC would lead to men having multiple sexual partners. This could be attributed to circumcised males who studies have shown to undergo a brief period of sexual experimentation shortly after circumcision [13, 28]. This practice could have led to the misconceptions held by males that MMC leads to men having multiple sexual partners. Other studies have reported significant differences in perceptions on sexual behaviours after MMC between males and females [27–29]. These provide important insights in the MMC decision making process. There was an association between being Muslim and the perception that MMC would increase the sexual drive of men. A study conducted in China concluded that circumcision decreases sexual enjoyment due to loss of nerve endings [8]. However, other studies have shown that MMC does not adversely affect sexual function in men [8, 30]. One common observation from these studies is that circumcised males experience changes in their sexual function. Changes in sexual enjoyment [8], penile sensitivity [30], and mean ejaculatory latency time [8] could explain the existence of this perception among Muslims. Pre-circumcision counseling should include messages about possible changes in sexual function and should address misconceptions related to sexual drive. This further emphasizes the importance of equipping communities with sufficient, accurate and consistent information about MMC. This study had some limitations. It investigated people's perceptions about the likely sexual behavioural outcomes of undergoing MMC and these might not necessarily and directly translate into post-circumcision practices. The study also relied on self-reported circumcision status which could have been misreported and may also be subject to socially desirable responses. Nevertheless, the study provides useful information on perceptions on MMC and related sexual behaviours to add to existing literature on use of the practice as part of the comprehensive package for HIV prevention as recommended by the World Health Organization.

## Conclusion

There were gaps in knowledge about MMC in this study. Negative perceptions about MMC and post circumcision sexual behaviours also exist in communities. These perceptions about the likely increase in the number of sexual partners, increase in sexual drive, reduced abstinence amongst youth and compromise of condom use could suggest the possibility of negative post circumcision sexual behaviours. To realize maximum benefits from MMC especially regarding HIV prevention and reduce possible instances of risk compensation, communities need to be equipped with sufficient, accurate and consistent information about the practice.
